# Deep learning structural insights into heterotrimeric alternatively spliced P2X7 receptors

**DOI:** 10.1007/s11302-023-09978-3

**Published:** 2023-11-30

**Authors:** Sophie K. F. De Salis, Jake Zheng Chen, Kristen K. Skarratt, Stephen J. Fuller, Thomas Balle

**Affiliations:** 1 Brain and Mind Centre, The University of Sydney, Camperdown, NSW 2050 Australia; 2 Sydney Pharmacy School, The University of Sydney, Camperdown, NSW 2050 Australia; 3 The University of Sydney, Nepean Clinical School, Kingswood, NSW 2747 Australia

**Keywords:** P2X7 receptors, Splice variants, AlphaFold2-Multimer, Models

## Abstract

P2X7 receptors (P2X7Rs) are membrane-bound ATP-gated ion channels that are composed of three subunits. Different subunit structures may be expressed due to alternative splicing of the *P2RX7* gene, altering the receptor’s function when combined with the wild-type P2X7A subunits. In this study, the application of the deep-learning method, AlphaFold2-Multimer (AF2M), for the generation of trimeric P2X7Rs was validated by comparing an AF2M-generated rat wild-type P2X7A receptor with a structure determined by cryogenic electron microscopy (cryo-EM) (Protein Data Bank Identification: 6U9V). The results suggested AF2M could firstly, accurately predict the structures of P2X7Rs and secondly, accurately identify the highest quality model through the ranking system. Subsequently, AF2M was used to generate models of heterotrimeric alternatively spliced P2X7Rs consisting of one or two wild-type P2X7A subunits in combination with one or two P2X7B, P2X7E, P2X7J, and P2X7L splice variant subunits. The top-ranking models were deemed valid based on AF2M’s confidence measures, stability in molecular dynamics simulations, and consistent flexibility of the conserved regions between the models. The structure of the heterotrimeric receptors, which were missing key residues in the ATP binding sites and carboxyl terminal domains (CTDs) compared to the wild-type receptor, help to explain their observed functions. Overall, the models produced in this study (available as supplementary material) unlock the possibility of structure-based studies into the heterotrimeric P2X7Rs.

## Abstract (summary)

AlphaFold2-Multimer was used to generate structures of the heterotrimeric P2X7 receptors composed of wild-type P2X7A subunits and alternatively spliced subunits (P2X7B, P2X7E, P2X7J, and P2X7L) that have been confirmed in humans. The study supports laboratory research by providing insight into the structure and flexibility of the heterotrimeric alternatively spliced receptors in a simulated environment and may thereby aid structure-guided drug design.

## Introduction

**Fig. 1:**
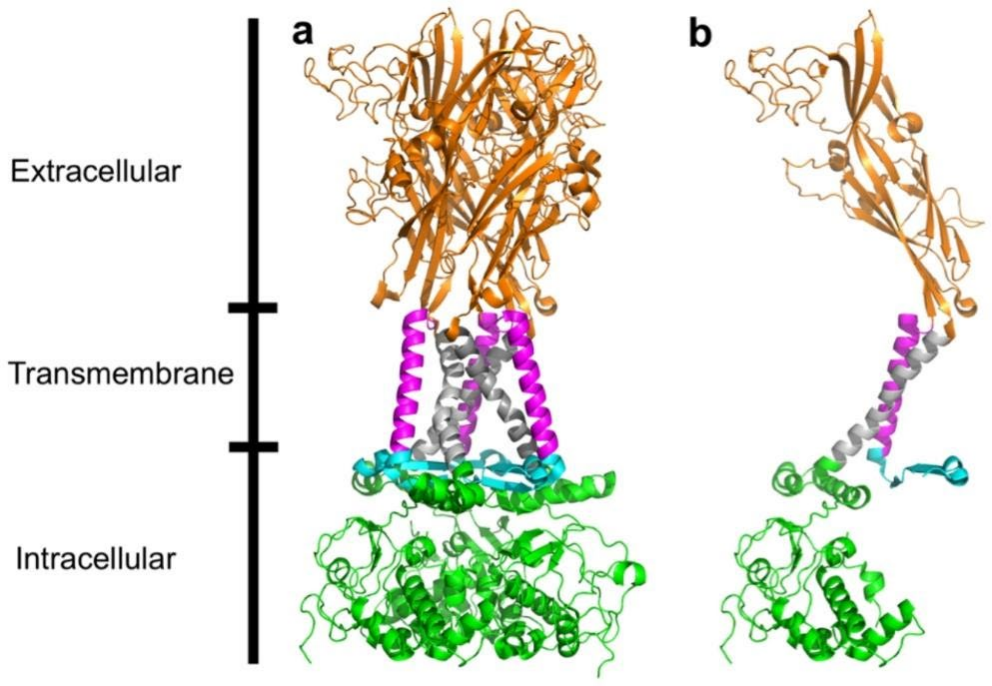
Apo closed state rat wild-type P2X7R structure determined by cryo-EM (Protein Data Bank Identification: 6U9V) [6] showing the amino terminal domain (cyan), transmembrane domain 1 (magenta), extracellular domain (orange), transmembrane domain 2 (grey), and carboxy terminal domain (green). **(A)** The trimeric P2X7R. **(B)** An individual P2X7R subunit. Figures were generated using the PyMOL molecular graphics system, version 2.5.3, Schrödinger, LLC.

**Fig. 2:**
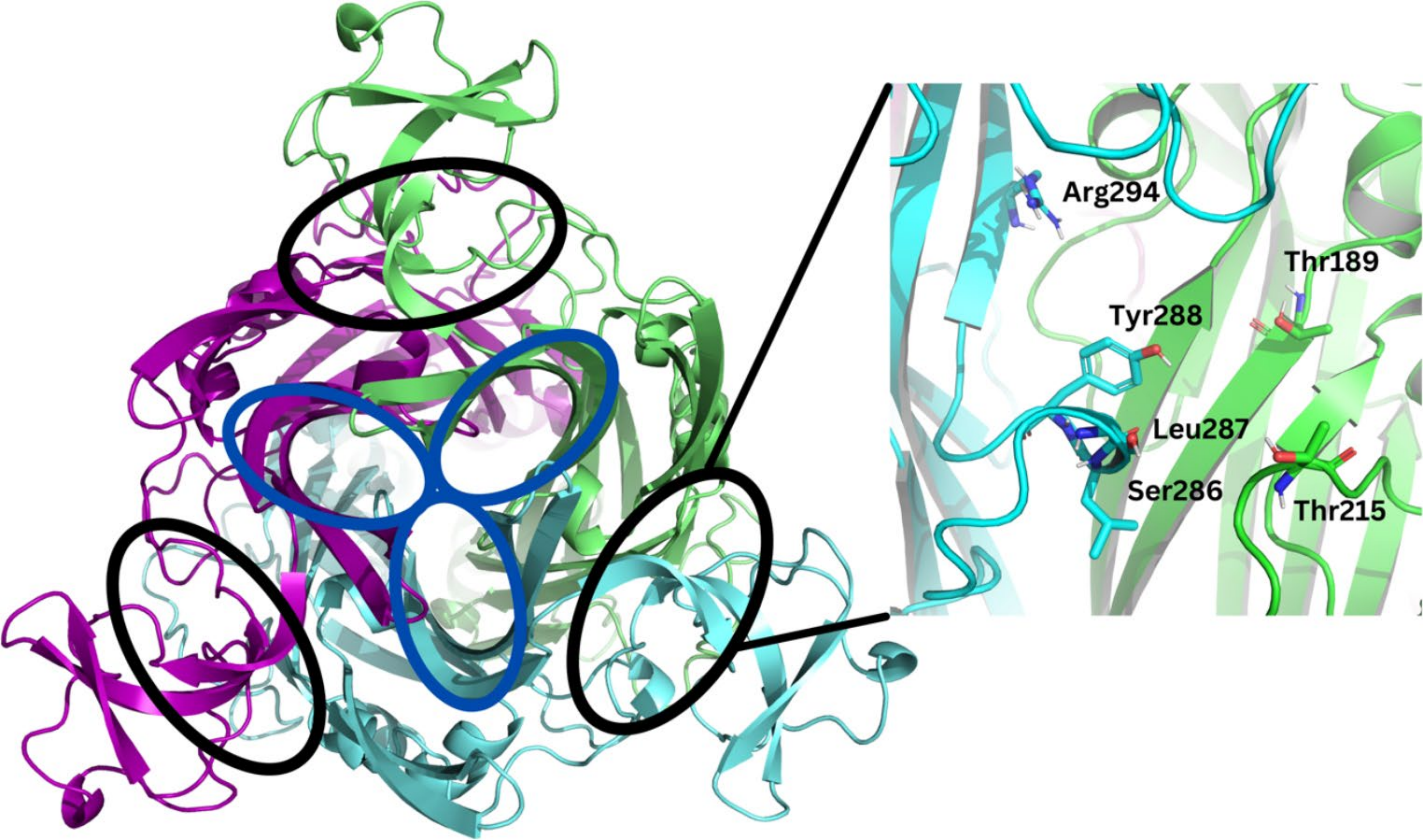
Birdseye view of the open state rat P2X7R determined by cryo-EM (Protein Data Bank Identification: 6U9W). The three subunits are coloured green, cyan, and magenta. The three ATP binding sites are circled in black. Each ATP binding site is located between two subunits (involving the key residues Thr189, Thr215, Ser286, Leu287, Tyr288, and Arg294) [6]. The three empty allosteric binding sites are circled in dark blue and are located within each subunit (involving Phe95, Phe103, Met105, Phe293, and Val312) [13–15]. The figure was generated using the PyMOL molecular graphics system, version 2.5.3, Schrödinger, LLC

P2X7 receptors (P2X7Rs) have been associated with cancers such as leukaemia [1], autoimmune conditions such as rheumatoid arthritis [2], trauma [3], and neurodegenerative conditions such as Alzheimer’s disease [4]. They are a subtype of the P2X receptors; a class of membrane-bound ATP-gated ion channels that are composed of three subunits (Fig. 1) [5]. Each P2X7R contains three ATP and three allosteric binding sites (Fig. 2) [6]. Activation by ATP results in the efflux of K^+^ and influx of Na^+^ and Ca^2+^ ions, enhancing cell proliferation and triggering a pro-inflammatory cascade [7]. It is widely supported that prolonged ATP activation correlates with cell apoptosis, however, the process through which this occurs is disputed. One theory suggests cell apoptosis occurs because the receptor’s channel diameter increases to form a pore that in vitro allows molecules up to 900 Da, such as YO-PRO-1 or ethidium bromide to pass through the cell membrane [8–10]. Riedel, et al. [11] disputes this pore formation theory, proposing that single channel kinetics and permeation properties are independent of receptor activation. Li, et al. [12], in turn, has suggested that cell apoptosis results from time-dependent alterations in intracellular ion concentrations due to shifts in equilibrium associated with prolonged P2X7R activation.

**Fig. 3:**
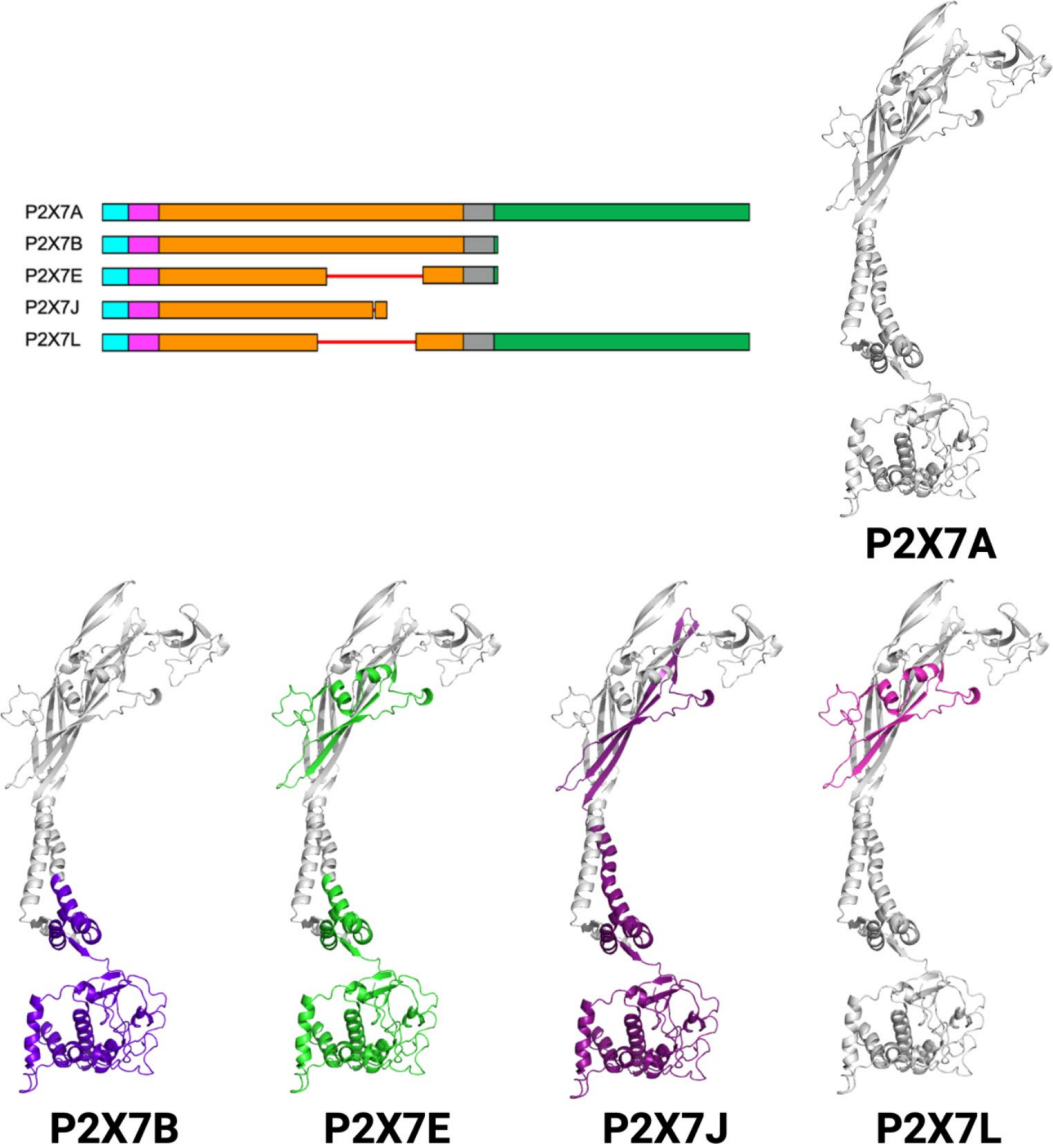
Schematic representation and cartoon representation of the wild-type P2X7A (UniProtKB: Q99572-1), P2X7B (UniProtKB: Q99572-2), P2X7E (UniProtKB: Q99572-5), P2X7J (GenBank: DQ399293.1), and P2X7L (GenBank: MK465687.1) subunit amino acid sequences generated using NCBI Multiple Sequence Alignment Viewer, version 1.22.0, National Center for Biotechnology Information, Rockville, MD, USA [19]. Residues 1 to 26 fold to form the intracellular ATD (blue), 27 to 51 the TMD 1 (pink), 52 to 330 the ECD (orange), 331 to 355 the TMD 2 (grey), and 356 to 595 the intracellular CTD (green) [20]. Regions of the protein missing are highlighted in purple (P2X7B), green (P2X7E), dark purple (P2X7J), and pink (P2X7L)

Alternative splicing of *P2RX7* messenger RNA (mRNA) results in the expression of different subunits that can associate to form different P2X7R trimers. Knowledge of the different combinations of P2X7 subunits that form P2X7R’s is key to supporting laboratory research and improving our understanding of the receptor’s role in disease. Five different subunits are currently known to be translated from the *P2RX7* gene (Table 1; Fig. 3) [16–18]. The primary structure of the wild-type P2X7A subunit consists of an intracellular amino terminal domain (ATD), transmembrane domain (TMD) 1, extracellular domain (ECD), TMD 2, and intracellular carboxy terminal domain (CTD) (Figs. 1 and 3) [8, 16]. Comparatively, P2X7B subunits lack the CTD [16]; P2X7E subunits are missing part of the ECD and CTD [18]; P2X7J subunits are missing part of the ECD, the entire TMD 2, and CTD [17]; and P2X7L subunits are missing part of the ECD [18].

**Table 1:** The five different subunits translated from the *P2RX7* gene in humans

Splice variants	Sequence length	Accession codes	
Wild-type P2X7A	595	UniprotKB: Q99572-1 [21]	
P2X7B	364	UniprotKB: Q99572-2 [16]	
P2X7E	275	UniprotKB: Q99572-5 [16]	
P2X7J	258	GenBank: DQ399293.1 [17]	
P2X7L	506	GenBank: MK465687.1 [18]	

Current research suggests P2X7B, P2X7E, P2X7J, and P2X7L subunits can associate with wild-type P2X7A subunits to form heterotrimeric receptors (P2X7A/A/B, P2X7A/B/B, P2X7A/A/E, P2X7A/E/E, P2X7A/A/J, P2X7A/J/J, P2X7A/A/L, and P2X7A/L/L receptors) [17, 18, 20]. ATP is more potent to P2X7B heterotrimers than wild-type P2X7A homotrimers [8]. P2X7J heterotrimers are dysfunctional whereby the P2X7J subunit may act as a negative regulator of P2X7R-induced apoptosis [17]. In co-transfection experiments a combination of P2X7A and P2X7E maintains P2X7R pore function however cells transfected with a combination of P2X7A and P2X7L have reduced pore formation capabilities in comparison to the wild-type P2X7A homotrimers [18].

P2X7B expression has been widely studied in cancer [7]. For example, a study observing P2X7R expression in myelodysplastic syndrome and acute myeloid leukaemia reported significantly higher wild-type P2X7A and P2X7B mRNA expression in acute myeloid leukaemia patients compared to those with myelodysplastic syndrome [22]. Furthermore, treatment-unresponsive patients were found to have lower levels of the wild-type P2X7A mRNA and higher P2X7B mRNA expression, bringing to question whether a P2X7B-selective antagonist could be beneficial in combination with current treatment regimens [22]. P2X7B has also been associated with cell proliferation in osteosarcoma [23] and neuroblastoma [24] making it an interesting focus for further research. P2X7J, despite its non-functionality, has been proposed as a potential biomarker for cervical cancer [17]. P2X7E and P2X7L currently have limited published information relating to disease associations but warrant further investigation based on their ability to express as functional proteins in combination with wild-type P2X7A subunits. For further information on P2X7R disease associations refer to De Salis, et al. [25]. It would be beneficial to study P2X7A, P2X7B, P2X7E, P2X7J and P2X7L isoform combinations to identify differences in their structures that may account for their different functions. Homomeric alternatively spliced receptors are largely insignificant due to deletion of a trafficking motif in the CTD that results in an inability to embed in the cell membrane (P2X7B, P2X7E, P2X7J), and altered ATP binding sites (P2X7L) preventing ion channel function. However, heterotrimeric alternatively spliced receptors warrant further investigation due to their observed functionality (P2X7B, P2X7E, P2X7L) and potential to highlight domains of the receptor that are crucial for functionality (P2X7J) [16–18].

Despite studies showing that the P2X7R plays a critical role in human disease, results of P2X7R antagonists in human trials have been disappointing. This is likely to have resulted from drug-target interaction studies that only considered the wild-type P2X7A receptor. The structures of P2X7R antagonists are diverse, indicating that in addition to the ATP binding site and an inhibitory drug-binding pocket [26], there are multiple other unmapped inhibitor binding sites. There is an opportunity to identify drug targets informed by the effects of alternative splicing considering the enormous promise of inhibiting P2X7Rs in human diseases. An exciting new deep learning-based modelling method, AlphaFold2-Multimer (AF2M) [27–29], has provided a pathway to elucidate these alternatively spliced structures. AF2M overcomes some of the limitations of traditional homology modelling which require high similarity templates to inform model generation [30]. AlphaFold has been shown to predict monomers in their lowest energy states with high accuracy, even when there is no equivalent structure in the PDB [28]. The subsequent version, AF2M, has been shown to successfully predict homomeric interfaces in 72% of cases and heteromeric interfaces in 70% of cases [27]. Homomeric/heteromeric models predicted by AF2M can be used as templates to aid the determination of experimental structures via molecular replacement in X-ray crystallography, and backbone tracing in cryo-EM [31].

This study primarily aims to validate the use of AF2M as a tool for P2X7R structure prediction, and then determine the structure of the alternatively spliced heterotrimeric receptors that express in humans in 2 A:1B/E/J/L and 1 A:2B/E/J/L stoichiometric ratios (P2X7A/(A/B)/B, P2X7A/(A/E)/E, P2X7A/(A/J)/J, and P2X7A/(A/L)/L receptors) to complement current in vitro research into heterotrimeric receptors’ structure and function. A secondary aim is to compare the heterotrimeric structures to identify structural differences that may account for their different functions. This study hypothesises that the unique structures of the heterotrimeric receptors can be modelled using AF2M and validated using AF2M’s confidence measures and molecular dynamics (MD) simulations.

## Materials and methods

The most recently published and highest quality experimentally obtained P2X7R structure is the apo closed state rat P2X7R determined by cryo-EM (Protein Data Bank Identification (PDB ID): 6U9V) [6]. Therefore, this structure was used as the experimental control for validation of the modelling approach. The residue and secondary structure numbering were assigned according to the wild-type P2X7A subunits. Refer to Table 2 for definitions on the validation criteria used and their cut-offs.

**Table 2:** Definitions of the validation criteria used for the modelled structures

Criteria	Definition	Values for a valid model
Predicted local distance difference test	An output from AF2M that represents the software’s confidence in the positions of the residues in the protein model out of 100	Values greater than 70 ^*a*^
Predicted template modelling score	An output from AF2M that represents the software’s confidence in the domain packing within the subunits of the protein model out of one. It is used in the ranking formula to identify the likely highest accuracy model	No specific cut-offs are available. The higher the number, the higher the confidence ^*b*^
Interface predicted template modelling score	An output from AF2M that represents the software’s confidence in the domain packing between the subunits of the protein model out of one. It is used in the ranking formula to identify the likely highest accuracy model	No specific cut-offs are available. The higher the number, the higher the confidence ^*b*^
Root mean square deviation (nm) with the experimental structure	A measure of the overall similarity between a computational structure and its equivalent experimental structure	Values less than 0.2 nm ^*c*^
Alignment score	A representation of the number of residues that align in the computational structure and its equivalent experimental structure	Values less than 0.7 ^*d*^
Template modelling score	A measure out of one of the similarities in protein folding of a subunit in the computational model compared to its equivalent experimental structure	Values greater than 0.6 have similar folding ^*e*^
Root mean square deviation (nm) in MD simulations	A measure of the overall stability of the receptor in a membrane system	Plateauing of the results over time ^*f−l*^
Root mean square fluctuation (nm)	A measure of residue-by-residue flexibility of the receptors. The higher the number, the higher the flexibility	Consistent regions of flexibility in similar models ^f−l^

^*a*^ [28]; ^*b*^ [27]; ^*c*^ [32]; ^*d*^ (Schrödinger Release_2022-3: Maestro, Schrödinger, LLC, New York, NY, 2021); ^*e*^ [33]; ^*f*^ [34]; ^*g*^ [35]; ^*h*^ [36]; ^*i*^ [37]; ^*j*^ [38]; ^*k*^ [39]; ^*l*^ [40]

### Method validation

AF2M was accessed as ColabFold 1.3.0 on the Australian National Computational Infrastructure Gadi supercomputer, using NVIDIA V100 graphics processing unit nodes (*gpuvolta* nodes) with 12 Intel Xeon Cascade Lake cores and 64GB of random-access memory per graphics processing unit [27–29]. The rat wild-type P2X7A subunit amino acid sequence (uniprotKB: Q64663) was retrieved from UniProt [41] and submitted as a full receptor (three wild-type P2X7A amino acid sequences were entered) into AF2M. The structure generation process was informed by completing three paired and unpaired multiple sequence alignments (MSA) on each of the three entered amino acid sequences, searching the UniRef100 and environmental sequences databases using Many-against-Many sequence searching [42–45]. Three .*a3m* MSA files were generated, one for each of the amino acid sequences. The files were manually combined into a single .*a3m* file for input into AF2M. 48 Evoformer blocks were used to process the MSA information with three ensemble iterations to produce final MSA and pair representations that were fed into eight structure module blocks to predict five protein structures and output a per-residue confidence score (the predicted local distance difference test (pLDDT)) (Table 3). The models were recycled through twenty iterative refinement cycles and relaxed using an AMBER protocol [46, 47] to improve the likelihood of obtaining accurate structures and to address potential AF2M issues by relaxing side chain conformations [27, 28]. The five models were ranked according to formula 1 [27].

**Table 3:** Interpretation of the pLDDT score generated by AF2M for each residue in the models [28]

pLDDT score	AF2M confidence
90–100	Very high confidence
70–89	High confidence
50–69	Low confidence
0–49	Very low confidence

$$Rank=\left(0.8 \times ipTM\right)+(0.2 \times pTM)$$

**Formula 1** The formula used to calculate the rank of the AF2M models. The predicted template modelling (pTM) scores and the interface pTM (ipTM) scores are measures of AF2M’s confidence in the domain packing within and between the receptor subunits respectively with scores ranging from zero (low confidence) to one (high confidence) [27].

The root mean square deviation (RMSD_experimental_) between the five ranked AF2M rat wild-type P2X7A receptor models and the experimental control were calculated using formula 2 via the Protein Structure Alignment function in Maestro (Schrödinger Release_2022-3: Maestro, Schrödinger, LLC, New York, NY, 2021) to measure their overall similarity.

$$RMS{D_{Experimental}}\left( {r,{r^{ref}}} \right) = \sqrt {\frac{1}{N}\sum\limits_{i = 1}^N {||{r_i} - r_i^{ref}|{|^2}} }$$

**Formula 2** Root mean square deviation between a computational model and its experimental structure (RMSD_experimental_) where N is the number of aligned residues, and r_i_ – r_i_^ref^ is the difference in the positions of the residue *i* in the AF2M models (r) compared to the experimental control (r^ref^) (Schrödinger Release_2022-3: Maestro, Schrödinger, LLC, New York, NY, 2021).

The template modelling (TM) scores of the AF2M models compared to the experimental control were calculated using formula 3 to measure the similarities in subunit folding [33, 48].

$$TM\,score = Max\left[ {\frac{1}{{{L_N}}}\sum\limits_{i = 1}^{{L_T}} {\frac{1}{{1 + {{\left( {\frac{{{d_i}}}{{ - 1.8 + 1.24\sqrt[3]{{({L_T} - 15)}}}}} \right)}^2}}}} } \right]$$

**Formula 3** The template modelling (TM) score between a computational model and its experimental structure where L_N_ is the length of the primary sequence of the AF2M subunits, L_T_ is the number of residues that correspond with the experimental control’s subunits, d_i_ is the distance between residue i in the AF2M models and its equivalent residue in the experimental control, and max represents the maximum d_i_ [33, 48].

ColabFold 1.3.0 does not have a native function to search for structural templates to inform the structure generation process. Thus, a custom template database was developed (containing the experimental structures of the apo closed state rat P2X7R (PDB ID: 6U9V) [6], giant panda P2X7R (PDB ID: 5U1L) [26], and zebrafish P2X4 receptor (PDB ID: 3I5D) [49]) and tested to determine the effect of using templates on the quality of the generated models. The RMSD_experimental_ of the top-ranked models generated with and without templates were subsequently compared.

### Heterotrimeric P2X7R models

The human wild-type P2X7A [21], P2X7B [16], P2X7E [16], P2X7J [17], and P2X7L [18] amino acid sequences were downloaded from the UniProt [41] and GenBank [50] databases. The method described above to model the rat P2X7R (with no custom template) was replicated to model the human wild-type P2X7A, P2X7A/(A/B)/B, P2X7A/(A/E)/E, P2X7A/(A/J)/J, and P2X7A/(A/L)/L receptors, altering the entered amino acid sequences to match the respective subunits. The top-ranked models for each of the heterotrimeric receptors were compared to the human wild-type P2X7A receptor model and their pLDDT scores were analysed.

### P2X7R molecular dynamics simulations

Receptor preparation was carried out using the Protein Preparation Wizard in Maestro (Schrödinger Release_2022-3: Protein Preparation Wizard; Epik, Schrödinger, LLC, New York, NY, 2021; Impact, Schrödinger, LLC, New York, NY; Prime, Schrödinger, LLC, New York, NY, 2021) [51]. The experimental control, human wild-type P2X7A, and heterotrimeric P2X7R models were pre-processed: assigned bond orders, aligned with the corresponding P2X7R structures in the Orientations of Proteins in Membranes (OPM) database [52, 53], and structural issues such as missing side chains and missing hydrogen atoms were corrected. Hydrogen bonds were optimised using PROPKA at pH 7.4 [54]. The heavy atoms and hydrogens were minimised using the OPLS4 force field [55] with a convergence threshold offset to 0.03 nm to address clashes that may have occurred from the addition of the side chains and hydrogens.

The experimental control is missing loops from residues 76 to 80, and 443 to 469 [6]. These were filled in automatically using Maestro Crosslink Proteins, predicting the loop conformation using simple *de novo* loop creation with an implicit solvent model in the energy calculation (Schrödinger Release_2022-3: Maestro, Schrödinger, LLC, New York, NY, 2021). The control was prepared again using Maestro’s Protein Preparation Wizard to address atom overlaps in the regions with added loops (Schrödinger Release_2022-3: Protein Preparation Wizard; Epik, Schrödinger, LLC, New York, NY, 2021; Impact, Schrödinger, LLC, New York, NY; Prime, Schrödinger, LLC, New York, NY, 2021) [51].

The receptor-membrane systems were prepared using a CHARMM36m force field in CHARMM-GUI [56–62]. Residues 360, 362, 363, 371, 373, 374, and 377 in the experimental control [6]; residues 4, 360, 362, 363, 374, and 377 in the human wild-type P2X7A and P2X7L subunits; residues 4, 360, 362, and 363 in the P2X7B and P2X7E subunits; and residue 4 in the P2X7J subunits were palmitoylated.

The receptors were inserted into lipid bilayers composed of 30% cholesterol [63, 64] (16% upper leaflet, 84 lipids; 14% lower leaflet, 70 lipids) and 70% 1-palmitoyl-2-oleoyl-sn-glycero-3-phosphocholine (POPC) (36% upper leaflet, 189 lipids; 34% lower leaflet, 170 lipids). The receptors and membranes were enclosed in rectangular systems (approximately 13 × 13 × 22 nm) with a water thickness above and below the receptor of 22.5 nm. Na^+^ and Cl^-^ ions were added to a concentration of 150 mM via random replacement of water molecules. Additional Na^+^ or Cl^-^ ions were added until the system charge was neutralised.

MD simulations were performed in GROMACS 2021.4 on NCI Gadi *gpuvolta* nodes using the CHARMM-GUI prepared systems to determine the stability of the AF2M-generated models in a POPC and cholesterol membrane system [34–40]. Minimisation, equilibration, and production steps were conducted using the same interaction and constraint algorithms as follows: particle neighbour lists with a neighbour search cut-off of 1.2 nm were updated every twenty steps during minimisation and every ten steps during equilibration and production; the particle pair interactions for each list were calculated using the Verlet cut-off scheme [65]; hydrogen bonds were defined as constraints using the LINCS algorithm [66]; and the interactions were constrained with a neighbour search cut-off of 1.2 nm using the Force-switch modifier [34–40] for van der Waal interactions and the fast smooth Particle-Mesh Ewald method [67] for electrostatic interactions.

Minimisation of the receptor-membrane systems adjusted the atomic arrangement to one that was energetically favourable (maximum force < 1000 kJ mol^-1^ nm^-1^) using the steepest descents method [68] with a maximum of 5000 steps. Six cycles of equilibration were performed to remove the centre of mass motion and equilibrate the receptor-membrane systems environmental factors using a time step of 2 fs. A Berendsen thermostat [69] was used to equilibrate the system at a constant temperature of 303.15 K with a coupling time constant of 1000 fs. A Berendsen barostat [69] was used to equilibrate the receptor-membrane system at a constant pressure of 1 bar and compressibility of 4.50e^-5^ bar^-1^ with semi-isotropic pressure coupling and a time constant of 1000 fs. Five randomly seeded production simulations were conducted for each of the systems using a time step of 2 fs and a total of 250,000,000 steps for a total simulation time of 500 ns. A Nosé-Hoover thermostat [70, 71] was used to maintain a constant temperature of 303.15 K with a coupling temperature fluctuation period of 1000 fs. A Parrinello-Rahman barostat [72] was used to maintain a constant pressure of 1 bar and compressibility of 4.5e^-5^ bar^-1^ with a semi-isotropic pressure coupling and a temperature fluctuation period of 5000 fs.

The simulation trajectories were post-processed using *gmx trjconv* in GROMACS 2021.4 [34–40] to centre the protein in the membrane system. The RMSD of the 2500 receptor frames (every 0.2 ns of the 500 ns production simulation) compared to the initial receptor frame (t = 0 ns) (RMSD_MD_) for each of the protein-membrane systems were calculated via *gmx rms* on GROMACS 2021.4 using formula 4.

$$RMS{D_{MD}}\left( t \right) = \sqrt {\frac{1}{{\text{M}}}\sum\limits_{i = 1}^n {{m_i}||{r_i}\left( t \right) - r_i^{ref}|{|^2}} }$$

**Formula 4** The root mean square deviation across a MD simulation (RMSD_MD_) where M is the sum of the atomic masses, N is the number of atoms, m_i_ is the mass of atom i and r_i_(t) is the position of atom i at time t in comparison to the position of atom i in the initial receptor conformation r^ref^ [34–40].

The root mean square fluctuation (RMSF) was calculated for each residue in the receptors via *gmx rmsf* in GROMACS 2021.4 [34–40] from 150 to 500 ns in the production simulation using formula 5.

$$RMSF\left( i \right) = \sqrt {\frac{1}{T}\sum\limits_{i = 1}^T {||{r_i}\left( t \right) - r_i^{ref}|{|^2}} }$$

**Formula 5** The root mean square fluctuation (RMSF) across a MD simulation where T is the number of frames included in the calculation, r_i_(t) is the position of residue i at time t and r_i_^ref^ is the position of the residue in the initial receptor conformation [34–40].

The generated models are available in the supplementary material.

**Fig. 4:**
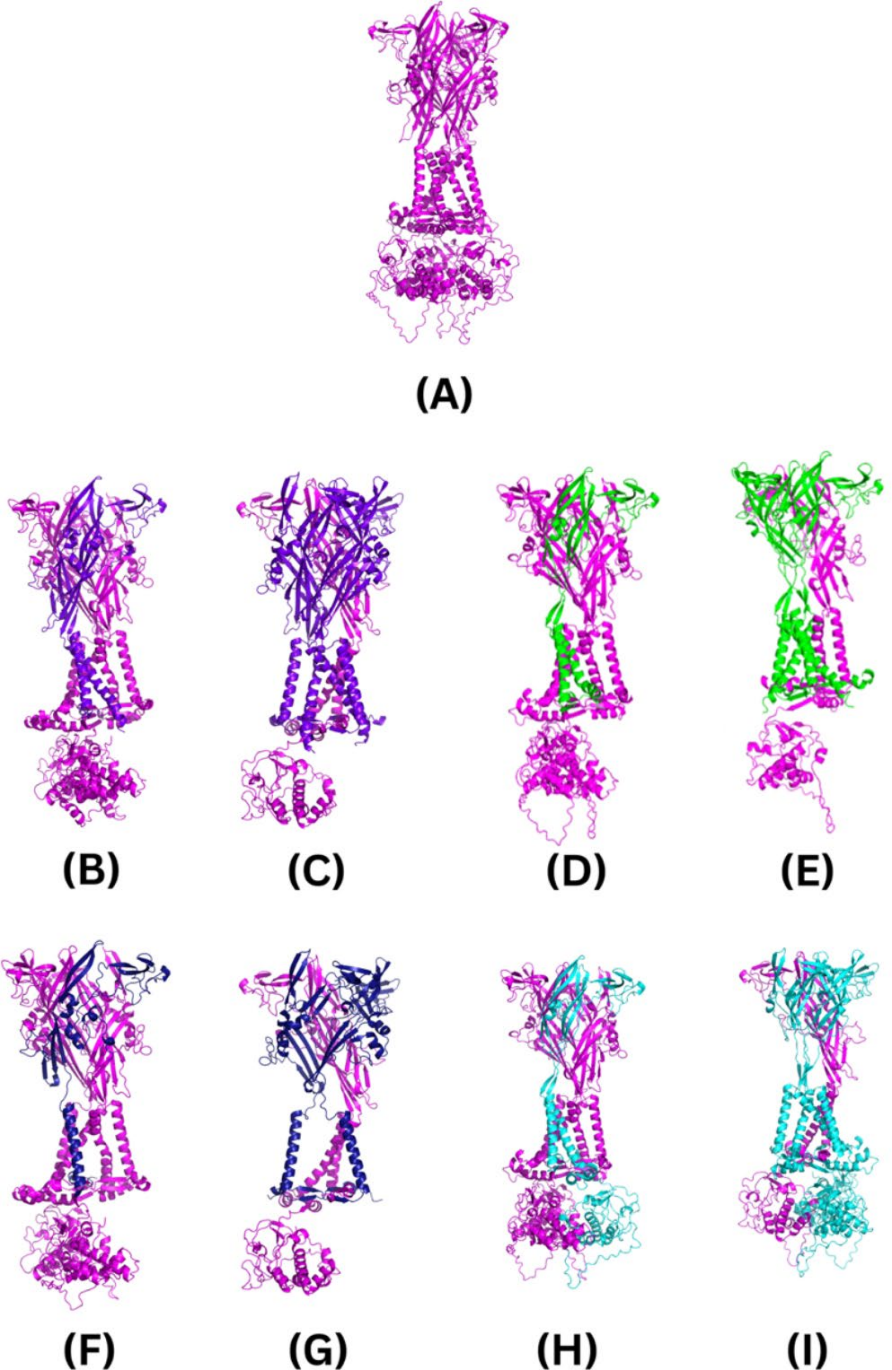
The AF2M-generated human P2X7R structures. The subunits are colour coded as follows: wild-type P2X7A is magenta; P2X7B is purple; P2X7E is green; P2X7J is dark blue; and P2X7L is cyan. **(A)** The wild-type P2X7A receptor; **(B)** P2X7A/A/B receptor; **(C)** P2X7A/B/B receptor; **(D)** P2X7A/A/E receptor; **(E)** P2X7A/E/E receptor; **(F)** P2X7A/A/J receptor; **(G)** P2X7A/J/J receptor; **(H)** P2X7A/A/L receptor; **(I)** and P2X7A/L/L receptor. Figures were generated using the PyMOL molecular graphics system, version 2.5.3, Schrödinger, LLC.

**Table 4:** The primary and secondary structures of the P2X7R subunits. The missing regions of the splice variants are defined as areas which are absent in comparison to the wild-type P2X7A subunit

Splice variant	Primary structures	Secondary structures - α helices	Secondary structures - β strands
Wild-type P2X7A	595 amino acids	22 α helices	21 β strands
P2X7B	364 amino acids (missing 231 residues from residue 365 to 595)	11 α helices (missing α12 to α22)	17 β strands (missing β18 to β21)
P2X7E	275 amino acids (missing 320 residues from residue 206 to 294 and 365 to 595)	7 α helices (missing α7 to α10, α12 to α22)	16 β strands (missing β12 to β15, β18 to β21, and β17 is split in two)
P2X7J	258 amino acids (missing 337 residues from residue 259 to 595)	10 α helices (missing α11 to α22)	11 β strands (missing β3, β7, β14 to β21)
P2X7L	506 amino acids (missing 89 residues from residues 206 to 294)	18 α helices (missing α7 to α10)	18 β strands (missing β12 to β15, and β17 is split in two)

## Results

### Method validation

The method was validated by comparing the AF2M-generated rat P2X7R model with the equivalent model obtained via cryo-EM (experimental control). The top-ranked rat P2X7R model (ranked based on its pTM = 0.78 and ipTM = 0.77) was predicted with high per-residue confidence with a pLDDT value of 79.40. Alignment of the top-ranked model with the experimental control revealed that the two structures were similar as demonstrated by an RMSD_experimental_ value of 0.18 nm, alignment score of 0.15, and a TM score of 0.96 (Table 2).

Observation of the AF2M ranking method revealed that the method could accurately identify the model with the highest similarity to the experimental control as the top-ranking method using the pTM and ipTM scores. Of the five generated rat P2X7R models, the top-ranked model had an RMSD_experimental_ of 0.18 nm from the experimental control compared to 0.37 nm, 0.26 nm, 0.30 nm, and 0.32 nm for rank 2, 3, 4, and 5 respectively.

The addition of a custom template database to inform the structure generation process did not impact the quality of the AF2M rat P2X7R models. The difference in RMSD_experimental_ of the top-ranked models with and without templates was 0.01 nm.

### Heterotrimeric P2X7R models

**Fig. 5:**
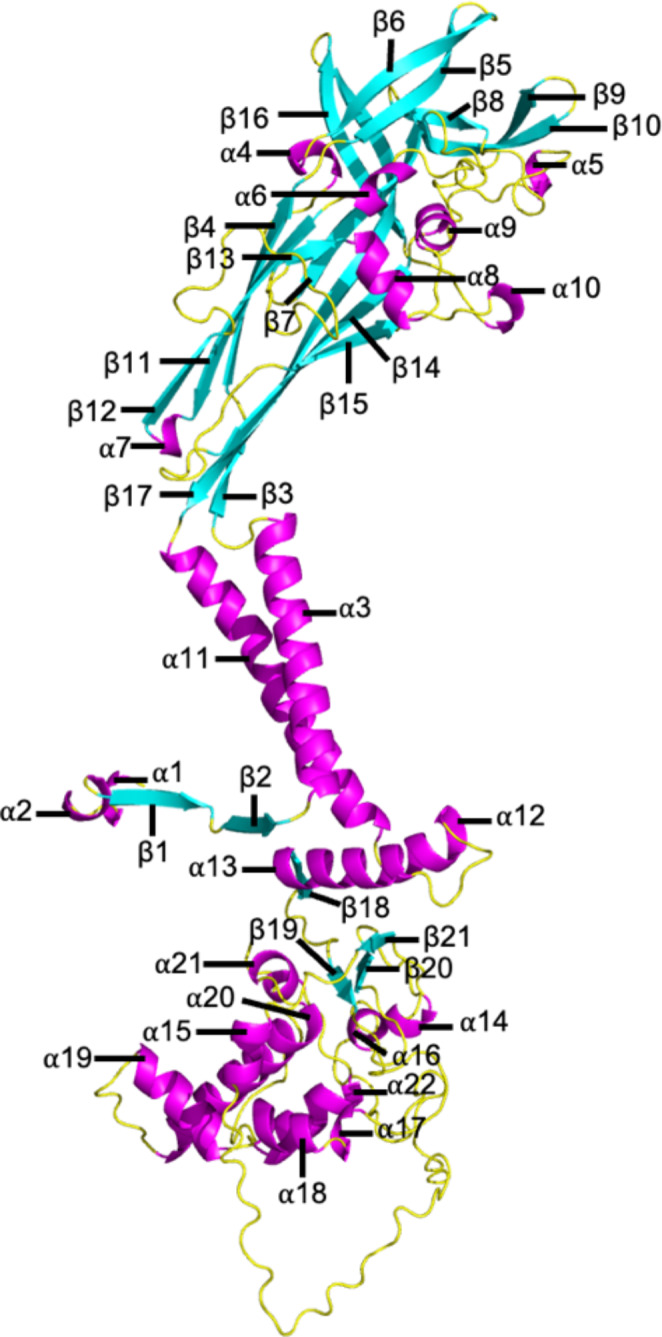
The AF2M-generated wild-type P2X7A subunit secondary structure with the naming of the individual segments adopted from McCarthy, et al. [6]. The figure was generated using the PyMOL molecular graphics system, version 2.5.3, Schrödinger, LLC.

The AF2M models of the top-ranked human wild-type P2X7A and heterotrimeric P2X7A/(A/B)/B, P2X7A/(A/E)/E, P2X7A/(A/J)/J, and P2X7A/(A/L)/L receptors are shown in Fig. 4. The elements of the wild-type P2X7A subunit secondary structure are outlined in Fig. 5 as a comparator for the splice variants. The primary and secondary structures of the subunits are outlined in Table 4.

**Fig. 6:**
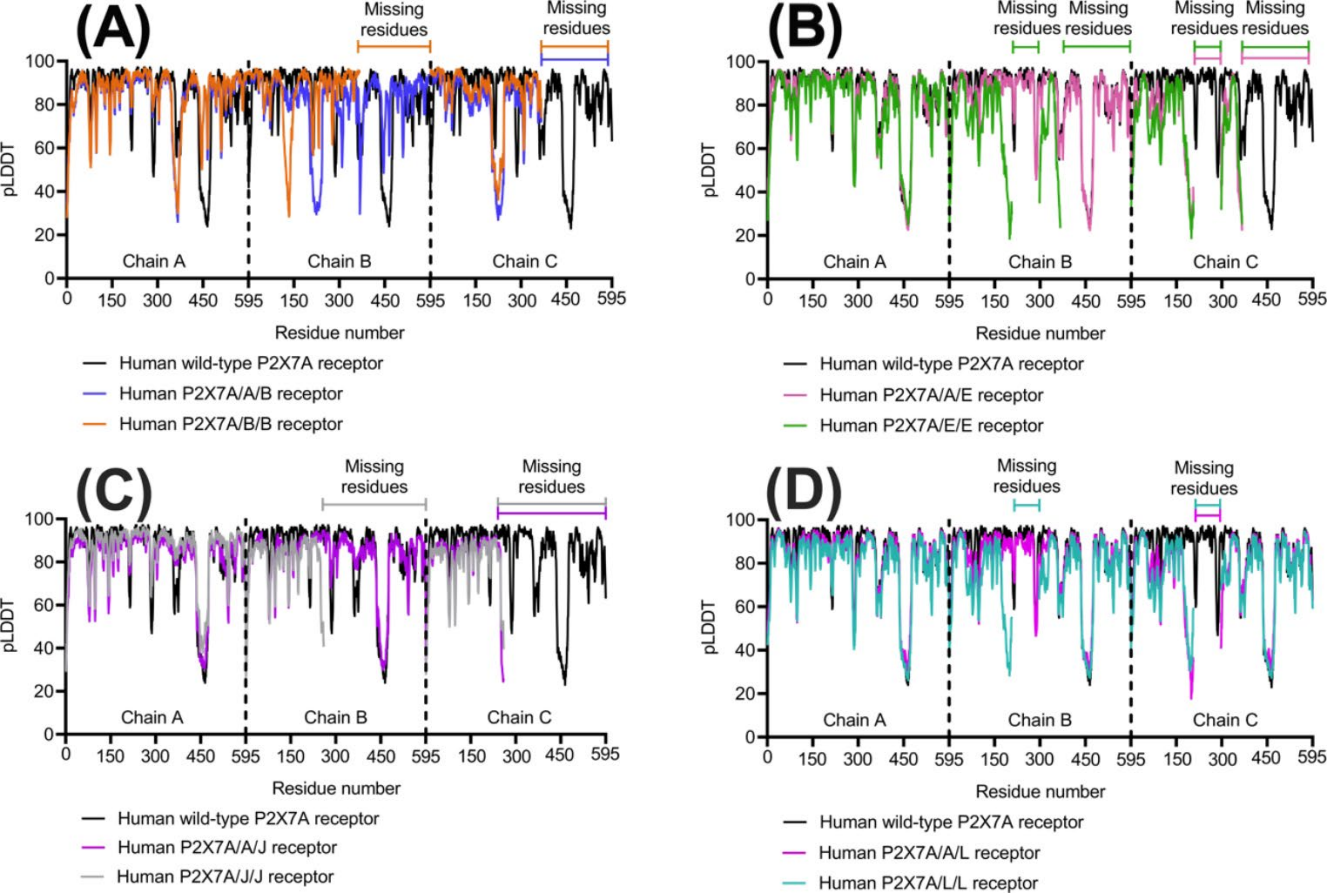
The wild-type homomeric P2X7A and heterotrimeric P2X7Rs pLDDT value per-residue out of 100 for each of the three subunits. The higher the pLDDT score, the higher the per-residue confidence. The heterotrimeric receptors compared to the wild-type P2X7A receptor **(A)** P2X7B **(B)** P2X7E **(C)** P2X7J **(D)** P2X7L. The graphs were generated using GraphPad Prism version 9.4.1 for MacOS, GraphPad Software, San Diego, California USA.

AF2M’s per-residue confidence score for each of the models was evaluated as a predicted measure of accuracy. All the top-ranked models were predicted with high per-residue confidence. The human P2X7A/B/B receptor was predicted with the highest confidence (pLDDT = 85.0), followed by the wild-type P2X7A (pLDDT = 83.5), P2X7A/J/J (pLDDT = 83.2), P2X7A/A/B (pLDDT = 81.8), P2X7A/A/J (pLDDT = 81.6), P2X7A/A/E (pLDDT = 81.0), P2X7A/A/L (pLDDT = 79.9), P2X7A/E/E (pLDDT = 76.1), and P2X7A/L/L (pLDDT = 76.1) receptors. Confidence in the model’s accuracy fluctuated on a per-residue basis as shown in Fig. 6. Lower per-residue confidence was observed in the loop regions of the receptors. For example, the loop region between β21 and α16 in the CTD (corresponding with residues 433 to 472) of the wild-type P2X7A and P2X7L subunits had a low per-residue confidence score (Fig. 6). Lower per-residue confidence was also observed in the loop regions near the spliced-out sites of the splice variants (residues 172 to 205, 295 to 303, and 349 to 365 in P2X7E; residues 249 to 258 in P2X7J; and residues 172 to 205, and 295 to 303 in the P2X7L splice variants) (Fig. 6).

### P2X7R molecular dynamics simulations

**Fig. 7:**
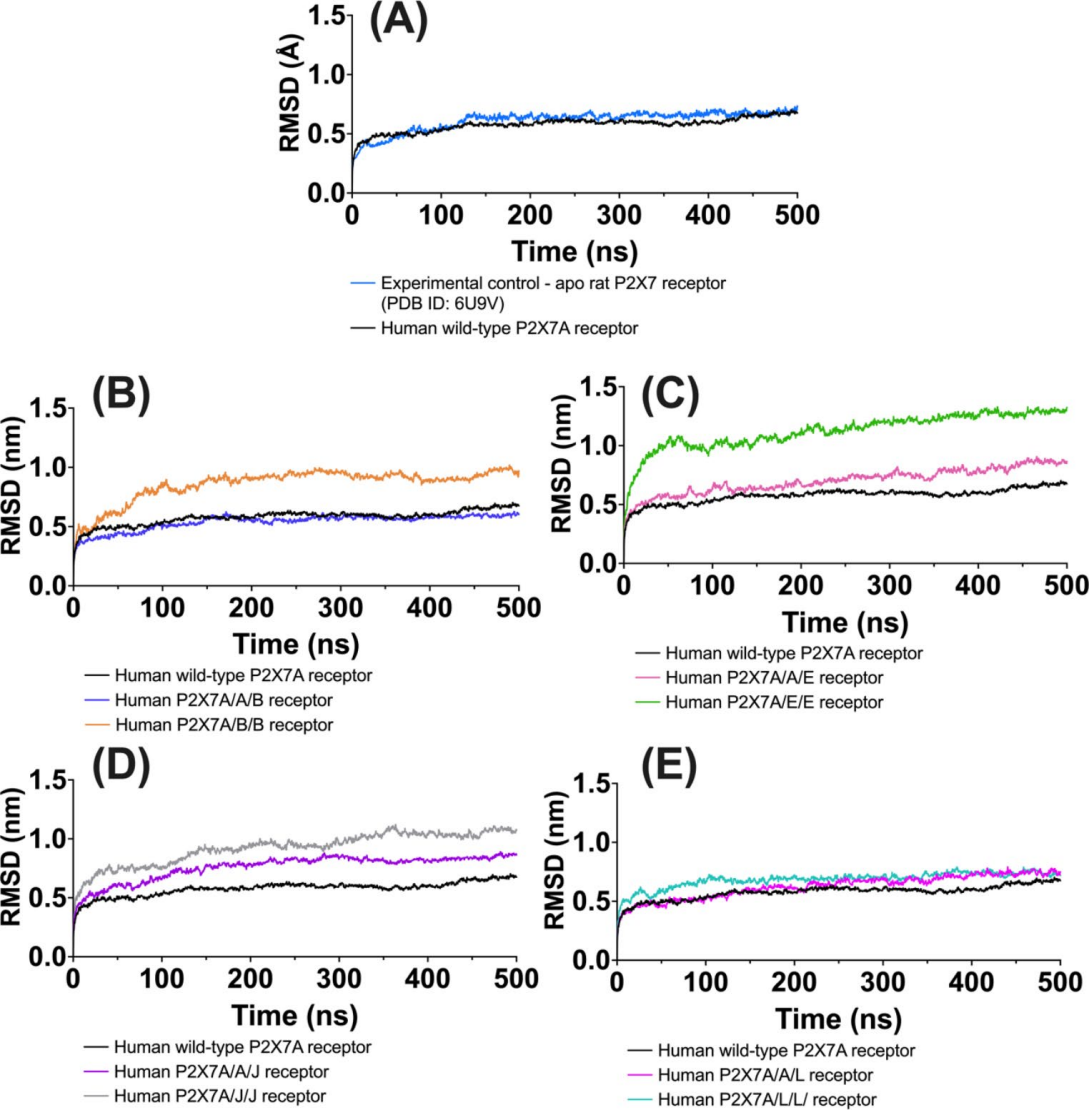
Average (n = 5) RMSD_MD_ (nm) over time (ns) for the human wild-type homomeric P2X7A and heterotrimeric P2X7Rs in comparison to their initial receptor conformation. Plateauing of the results suggests the receptor is stabilising in the MD simulation trajectory. **(A)** The experimental control (rat P2X7R) and human wild-type P2X7A receptor model. Human wild-type homomeric P2X7A receptor compared to heteromeric receptors comprising P2X7A and: **(B)** P2X7B, **(C)** P2X7E, **(D)** P2X7J, **(E)** P2X7L, in a 2:1 and 1:2 stochiometric ratio. The graphs were generated using GraphPad Prism version 9.4.1 for MacOS, GraphPad Software, San Diego, California USA.

MD simulation outputs were observed to identify the stability and flexibility of the receptors in a simulated membrane system. Similar stabilities were observed between the experimental control and the AF2M-generated human wild-type P2X7A and heterotrimeric receptors as shown in the RMSD_MD_ outputs. The RMSD_MD_ outputs increased within the first 1 ns of the simulations following the introduction of the initial particle velocities to the systems (Fig. 7). The outputs then plateaued to a mean *±* standard deviation from 150 to 500 ns in the simulation (with 150 ns as the baseline) of 0.66 *±* 0.02 nm (0.04 nm) for the experimental control compared to 0.61 *±* 0.03 nm (0.04 nm) for the human wild-type P2X7A, 0.57 *±* 0.02 nm (-0.02 nm) for the P2X7A/A/B, 0.93 *±* 0.03 nm (0.04 nm) for the P2X7A/B/B, 0.75 *±* 0.07 nm (0.11 nm) for the P2X7A/A/E, 1.20 *±* 0.08 nm (0.14 nm) for the P2X7A/E/E receptors, 0.82 *±* 0.03 nm (0.04 nm) for the P2X7A/A/J, 1.00 *±* 0.06 nm (0.08 nm) for the P2X7A/J/J, 0.68 *±* 0.05 nm (0.09 nm) for the P2X7A/A/L and 0.71 *±* 0.03 nm (0.02 nm) for the P2X7A/L/L (Fig. 7).

**Fig. 8:**
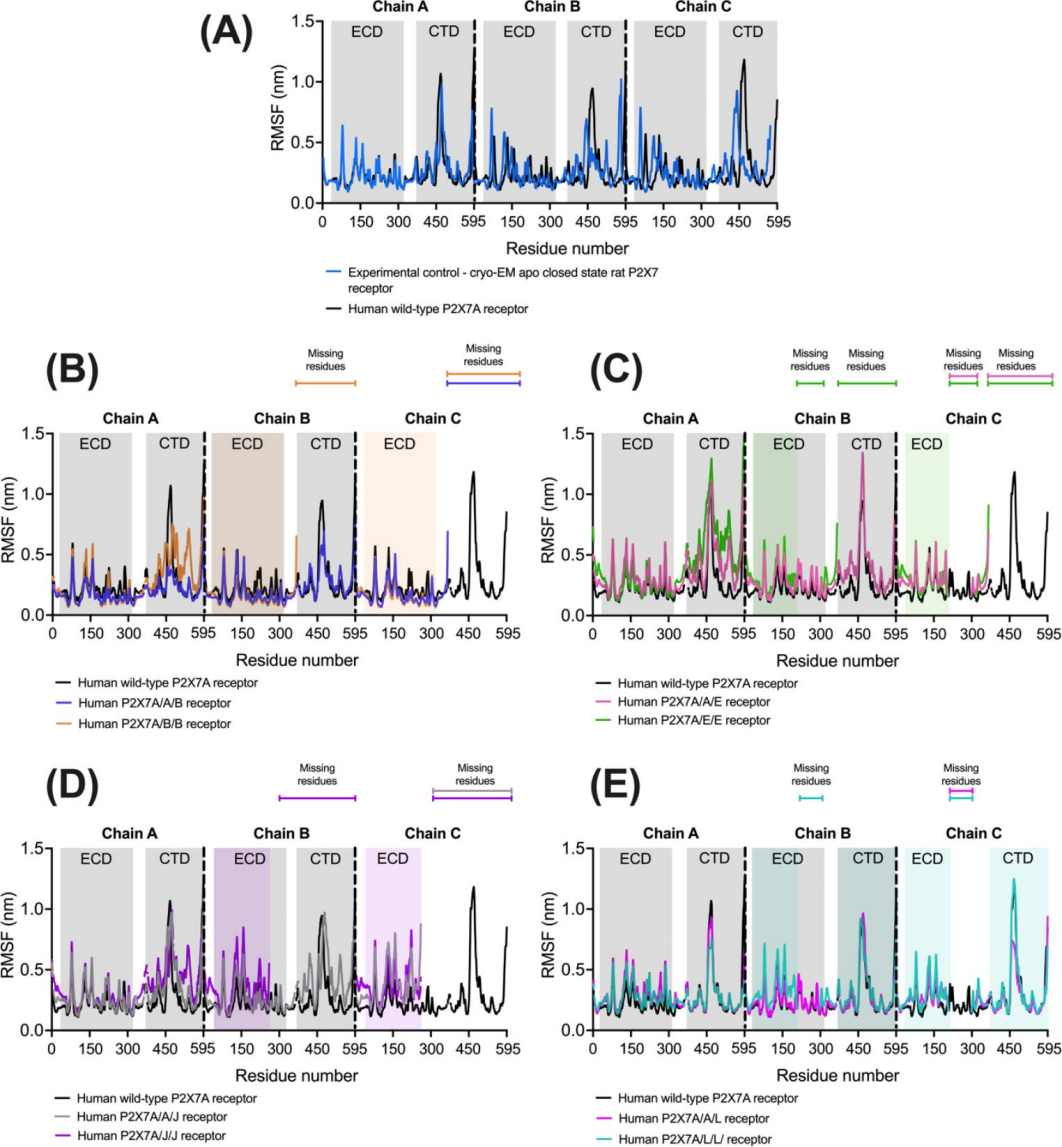
The wild-type homomeric P2X7A and heterotrimeric P2X7 receptor’s RMSF (nm) per residue for each of the three subunits (chain A, B, C). Peaks represent regions of flexibility. The results were obtained from the MD simulations. **(A)** The experimental control and human wild-type P2X7A receptor model. The heterotrimeric receptors compared to the wild-type P2X7A receptor **(B)** P2X7B **(C)** P2X7E **(D)** P2X7J **(E)** P2X7L. The graphs were generated using GraphPad Prism version 9.4.1 for MacOS, GraphPad Software, San Diego, California USA.

The flexibility of the residues in the receptors were consistent between the experimental control and the human wild-type P2X7A receptor AF2M-generated structure as shown in the RMSF outputs (Fig. 8A). A macroscopic inspection of the RMSF outputs for the experimental control and all the AF2M-generated structures identified peaks in flexibility in the loop segments of the receptors (Fig. 8). The highest RMSF peaks were observed in an unstructured region of the CTD in the wild-type P2X7A and P2X7L subunits from residues 446 to 492 (the loop between α9 and α10) that has not been elucidated in the rat P2X7R cryo-EM structure (Fig. 8E).

The heterotrimeric receptors consisting of two splice variants were notably more flexible than those containing only one splice variant. In particular, the remaining intracellular regions of the P2X7A/B/B, P2X7A/E/E, and P2X7A/J/J heterotrimeric receptors were more flexible, consistent with these receptors being less restricted due to the absent large CTD that is present in the wild-type P2X7A and P2X7L subunits (Fig. 8A-E). The splice variants also reported higher flexibilities in comparison to the wild-type P2X7A subunits (Fig. 8A) in the residues located near the spliced-out sites (residues 358 to 364 in the P2X7B subunits (Fig. 8B); residues 117 to 203, 300 to 306 and 320 to 325 in the P2X7E subunits (Fig. 8C); residues 254 to 258 in the P2X7J subunits (Fig. 8D); and residues 117 to 203 and 300 to 306 in the P2X7L subunits) (Fig. 8E).

### Model analysis

**Fig. 9:**
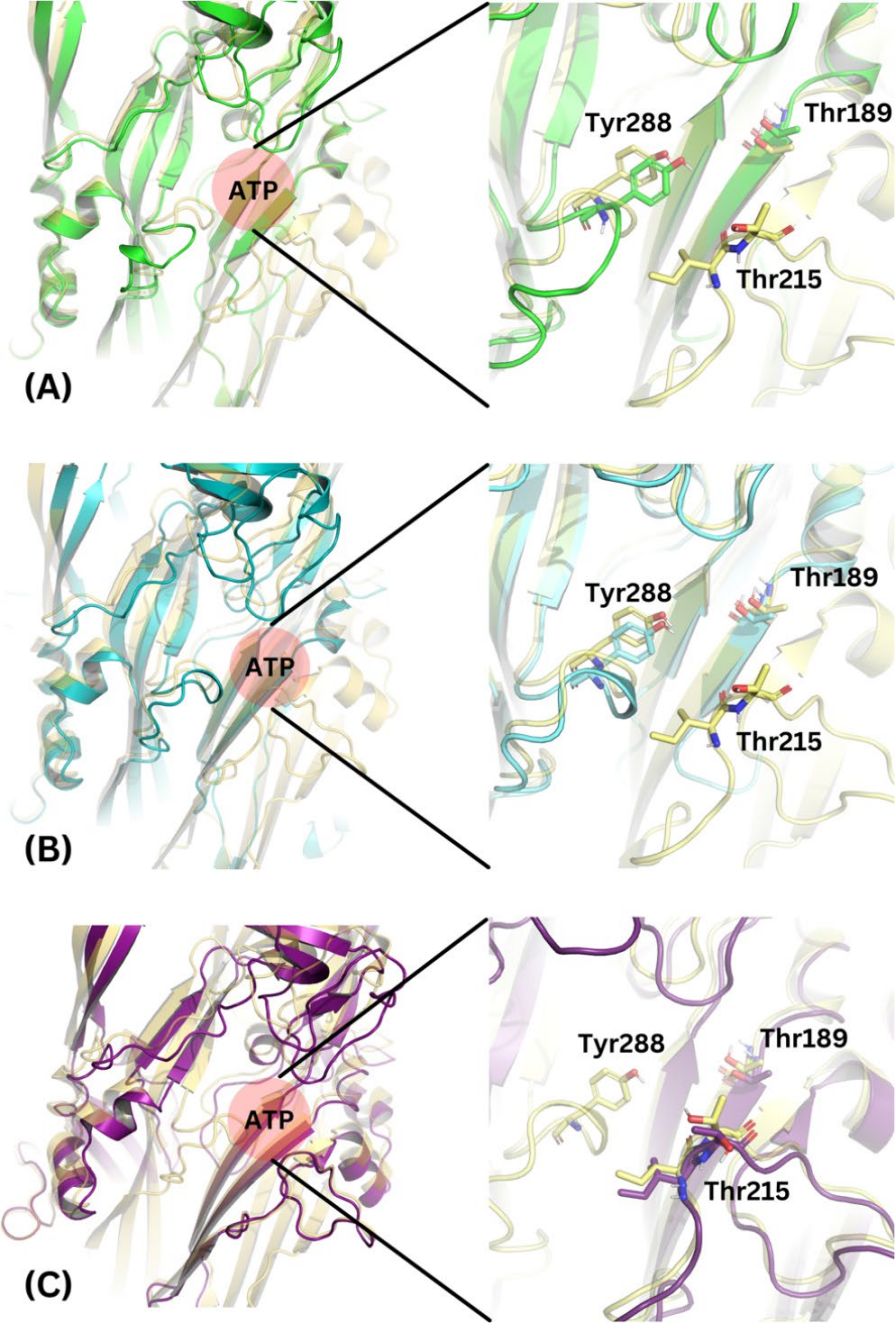
Model analysis of the sites of interest in the AF2M-generated P2X7R models. **(A)** ATP binding regions in the wild-type P2X7A (yellow) and P2X7E (green) subunits. **(B)** ATP binding regions in the wild-type P2X7A (yellow) and P2X7L (cyan) subunits. **(C)** ATP binding regions in the wild-type P2X7A (yellow) and P2X7J (dark purple) subunits. **(D)** Cartoon schematic of the ATP binding region demonstrating the three “lobes” of the region with their corresponding residue numbers, ATP (red) is shown in the centre. Residues Thr189, Thr215, and Tyr288 are shouwn in stick representation

The P2X7E, P2X7J, and P2X7L splice variants with known modifications to the ATP binding region were further analysed. P2X7E and P2X7L are both missing the ATP binding regions from residues 206 to 294. P2X7J is missing all residues after residue 258. In the P2X7R models containing P2X7E (Fig. 9A), P2X7L (Fig. 9B), or P2X7J subunits (Fig. 9C), the ATP binding interfaces are disrupted. Different residues are located at the ATP binding region in each of the splice variants because of their altered structures, and residues that are the same have higher flexibilities in the splice variants compared to the wild-type P2X7A isoform (Table 5). The ATP binding sites in the P2X7E and P2X7L splice variants compared to the wild-type P2X7A subunits revealed that a key part of the ECD that forms the outer “lobe” of the ATP binding region is missing, while the inner two “lobes” of the binding region remain intact (Fig. 9C). P2X7J splice variants are missing the inner “lobes,” modifying the ATP binding site in a different manner. Accordingly, the P2X7A/A/E, P2X7A/A/L, and P2X7A/A/J receptors have one disrupted ATP binding site while the P2X7A/E/E, P2X7A/L/L, and P2X7A/J/J receptors have two disrupted ATP binding sites. The palmitoylated side chains behaved uniformly across the simulations.

**Table 5:** Residues within 6 Å of ATP in the main binding pocket for each of the isoforms and their corresponding RMSF (nm)

	Rat P2X7A	Human P2X7A	P2X7B	P2X7E	P2X7J	P2X7L
Lys66	0.17	0.14	0.14	0.24	0.23	0.26
Arg126						0.37
Pro142						0.22
Gln143	0.21	0.21	0.25	0.28	0.46	0.22
Thr189	0.17	0.15	0.15	0.28	0.24	0.28
Leu191	0.14	0.15	0.13	0.34	0.22	
Ile214	0.30	0.32	0.32		0.46	
Thr215	0.27	0.28	0.30		0.48	
Cys216					0.51	
Ile228	0.18	0.18	0.16		0.28	
Ser286	0.34	0.34	0.21			
Leu287	0.28	0.32	0.18			
Tyr288	0.24	0.25	0.16			

## Discussion

This study provides insight into the previously unknown structures of the human wild-type P2X7A, P2X7A/(A/E)/E, P2X7A/(A/B)/B, P2X7A/(A/J)/J, and P2X7A/(A/L)/L receptors derived using a deep learning predictive modelling method, AF2M. The models were validated and analysed using AF2M’s confidence measures and MD simulations, with the aim of providing templates for further P2X7R structure-function related research and drug discovery. Furthermore, the computational models can be used to aid the elucidation of future cryo-EM or X-ray crystallographic structures as highlighted by Kryshtafovych, et al. [31] using computational models as a template for molecular replacement in X-ray crystallography, and backbone tracing in cryo-EM [31].

### Method validation

The computational modelling of protein structures has piqued the interest of researchers over the past three decades with biennial Critical Assessment of Structure Prediction (CASP) competitions run since 1994 [73]. The release of AlphaFold by the DeepMind group in 2021 enabled the prediction of protein structures to an experimental quality based on their amino acid sequence alone [28]. The DeepMind group subsequently released an artificial intelligence modelling method, AF2M, that could predict the structures of protein complexes containing more than one subunit [27]. As AF2M is a novel protocol, there are limited published applications and validations of the method under different conditions and settings. Thus, in this study, we first investigated whether P2X7Rs, that are large receptor complexes, could be successfully modelled using AF2M. The number of times each of the models were recycled through iterative refinement cycles was increased from the default of three to twenty. As Wallner [74] observed, increasing the number of sampled models improves the quality of the predictions. CASP14 competition criteria were used to validate the method (RMSD_experimental_, alignment score, and TM score) [75]. The measures suggested the top-ranked AF2M-generated rat P2X7R model is a valid representation of the experimentally determined closed receptor conformation based on an RMSD_experimental_ value of less than 0.2 nm [32], alignment score of less than 0.7 (Schrödinger Release_2022-3: Maestro, Schrödinger, LLC, New York, NY, 2021) and TM score of greater than 0.6 [33].

The TM score (actual value) is 0.18 higher than the pTM score (predicted value). This result supported findings by Jumper, et al. [28] that suggested the pTM is a conservative estimate of the TM score as it is based on the lower bounds of the pairwise matrix (a matrix used to identify the positional errors of the α-carbons in the structures) and therefore should be used as a comparator between the AF2M models through ranking the models from highest to lowest quality, rather than a definitive measure [27, 28]. The RMSD_experimental_ was used to evaluate the ranking method of AF2M, which uses the pTM and ipTM scores. The top-ranking model has the highest similarity to the experimental control, supporting the decision to select the top-ranked model for further study when considering the AF2M-generated heterotrimeric receptors.

As part of the method validation process, the addition of a custom template database was investigated. The AF2M models produced when a custom template database was used to inform the structure generation process were comparable to those produced when no template database was used. Originally, it was hypothesised that the custom templates may improve the predictions as they are integral to the original AlphaFold release [28]. However, based on the results the quality of the AF2M predictions was likely dependent on the quality of the MSA representations rather than the templates as supported by Evans, et al. [27].

### Heterotrimeric receptors

The structures of P2X7Rs that incorporate splice variants in combination with one or two wild-type P2X7A subunits were modelled. These structures present a challenge to traditional homology modelling methods as they are missing large domains inside the protein sequence. Deep learning methods such as AF2M can overcome this limitation of homology modelling. Visual inspection of the structures, AF2M’s confidence measures, and MD simulation outputs (RMSD_MD_ and RMSF) were used to determine the quality of the AF2M models. These measures were used as currently there are no experimentally obtained structures available for the splice variants.

Overall, the top-ranking models were predicted with high confidence in relation to the pLDDT output, suggesting the models are good predictions of the protein backbone [28]. The structural irregularity of loop regions [76] and the reduced number and sequence similarities of MSAs (that inform the structure generation process) [29] can account for some of the decreases in pLDDT, in particular from residues 433 to 472 in the P2X7A and P2X7L subunits. Residues 433 to 472 are a loop region in the CTD not observed in any of the other six P2X receptors [77]. The reduction in pLDDT in residues near spliced-out sites in the receptors can be explained by their reduced inter-residue contacts that are key to informing the structure generation process. This is supported by the findings of Jumper, et al. [28] that suggested higher numbers of inter-residue contacts are linked with higher prediction confidence scores. The important zinc binding site present within the CTD is found to be conserved in the P2X7L splice variant as compared to the wild-type P2X7A.

MD simulations were used as an alternate method to validate the AF2M structures in a membrane system, a validation method that has previously been used in the literature to confirm the stability of models [78–80]. The RMSD_MD_ initially increased to > 0.5 nm over the first 150 ns of the simulations following the introduction of the initial particle velocities to the systems. The RMSD_MD_ then plateaued from 150 to 500 ns as the models re-arranged to a stable formation. The RMSF outputs of the predicted models are consistent with the experimental control, highlighting similar regions of flexibility (predominantly in the loop regions), although to different extents, supporting the validity of the models. Higher flexibility was observed in the loop regions, in particular in the CTD and ECD exposed to the intracellular and extracellular environment.

### Structure-function analysis

#### P2X7B

The predicted models help to explain the previously identified functions of the alternatively spliced P2X7Rs. P2X7B homotrimers are reported to be non-functional, Masin, et al. [81] hypothesised this is due to their inability to traffic to the cell membrane. These homotrimers are missing the three CTDs that are native to the wild-type receptor. The CTDs contain motifs that are associated with pore dilation (cytoplasmic ballast), phagocytosis (sarcoma tyrosine kinase homology 3 (SH3) domain, cell death (TNF receptor 1 death domain), and membrane binding (membrane protein-cytoskeleton linking domain and palmitoylated cysteine rich domain) [25].

Compared to homomeric P2X7B receptors, experimental data suggests P2X7A/B heterotrimers are functional with increased ion channel function and pore formation compared to the wild-type P2X7A receptor [20]. The AF2M-predicted structure of P2X7A/B heterotrimeric receptors may explain the enhanced receptor function observed in vitro. The models show that the ATP binding site residues and flexibilities are equivalent to those of the wild-type P2X7A receptor (Table 5). The significant difference between the heterotrimeric receptors and the wild-type P2X7A receptor is that the P2X7A/A/B and P2X7A/B/B receptors are missing two and one CTDs respectively. The flexibilities of the two CTDs in the P2X7A/A/B (average CTD RMSF = 0.34 and 0.42 nm) are similar to the wild-type P2X7A receptor (average CTD RMSF = 0.33 nm, 0.31 nm, and 0.32 nm). However, the flexibility of the CTD in the P2X7A/B/B receptor (average CTD RMSF = 0.73 nm) is significantly higher than the equivalent CTD in the wild-type P2X7A receptor (average CTD RMSF = 0.33 nm) (Fig. 8B). The remaining CTD in the P2X7A/B/B receptor likely allows the receptor to traffic to the cell membrane, maintaining the membrane ion channel functionality [25] while the two missing CTDs increase the channel accessibility and could account for the potentiation in receptor function observed by Adinolfi, et al. [20].

#### P2X7E

Homomeric P2X7E receptors, missing the CTD and part of the ECD, are also non-functional [18]. The missing CTDs likely prevent these receptors from trafficking to the cell membrane [81]. Compared to the homomeric P2X7E receptors, Skarratt, et al. [18] observed the same function with heterotrimeric P2X7A/E receptors as with wild-type P2X7A receptors. The predicted P2X7A/A/E and P2X7A/E/E receptor models can help to explain this phenomenon where the receptors are missing one and two CTDs and have one and two altered ATP binding sites respectively (Table 5). Gusic, et al. [82] suggested that although three ATP molecules will bind preferentially, a single ATP molecule is sufficient to evoke channel activation, but activation will occur at a reduced speed. The in vitro findings of similar function in P2X7A/E receptors compare to wild-type may result from a potential increase in receptor function due to the flexible CTD in the P2X7A/E/E receptor (Fig. 8C) counteracting the potential reduction in function associated with reduced ATP binding.

#### P2X7J

The homomeric P2X7J receptor is unable to traffic to the cell membrane and is consequently non-functional [17]. Comparatively, Feng, et al. [17] suggested P2X7J antagonises P2X7A subunit activity including cell apoptosis. The missing CTD in the P2X7J isoform, similarly to the P2X7B and P2X7E isoforms, likely prevents the homomeric P2X7J receptor from embedding in the cell membrane. In relation to the heterotrimeric P2X7A/J receptors, the P2X7J isoform is missing residues 259 to 595, including Ser286, Leu287 and Tyr288 that are in the wild-type P2X7A ATP binding sites (Table 5). The missing components in the ATP binding domain of the P2X7J variant suggests that this subtype is unlikely to bind ATP. The P2X7A/J and P2X7J/J interfaces appear identical as the constituent of the ATP binding site consisting of Thr189 and Thr215 is conserved in P2X7A and P2X7J. There is however the loss of the binding residues Ser286, Leu287, and Tyr288, which is not present in the wild-type P2X7A/A interface. The RMSF fluctuates higher and more frequently in the heterotrimeric P2X7J receptors residues than in the wild-type receptor (Fig. 8D). In the MD simulations, the P2X7A ECDs leans towards the P2X7J isoforms. The P2X7J isoforms appear to lack the structural integrity to maintain the upright receptor conformation, likely because of the missing TMD 2. The combination of the missing residues in the ATP binding sites and the reduced stability of the receptor may explain the lack of pore formation and apoptotic activity on exposure to benzoyl-ATP observed in heterotrimeric P2X7A/J receptors by Feng, et al. [17]. Further research on P2X7A/J receptor expression is required to support this hypothesis.

#### P2X7L

Experimental data obtained by Skarratt, et al. [18] found that homomeric P2X7L receptors have no ion channel or pore function but still have phagocytic function. The unchanged CTD (containing the SH3 domain that is required for phagocytic function [25]) in the predicted P2X7L isoforms explains the observed phagocytic function while the lack of ion channel function suggests the ATP binding sites are non-functional. The heterotrimeric P2X7A/L receptors are reported to have ion channel function as well as phagocytic function, although to a lesser extent than the wild-type P2X7A receptor [18]. The reduced ion channel function in the heterotrimeric A/L receptors observed by Skarratt, et al. [18] can be explained by the likely reduction in the number of ATP binding sites. The P2X7A/A/L and P2X7A/L/L receptors have two and one ATP binding sites that resemble those seen in the wild-type P2X7A receptors. Compared to P2X7A, P2X7L isoforms are missing key residues in the ATP binding site (Leu191, Ile214, Thr215, Ile228, Ser286, Leu287 and Tyr288) and additional residues (Arg127 and Pro142) extend into the binding site (Table 5). The residues surrounding the ATP binding site have higher flexibility in comparison to the wild-type P2X7A receptor (Fig. 8E), that are likely to impede ATP binding.

#### Limitations

It is important to acknowledge that the structures are predictions rather than experimental structures and therefore should be continuously validated as new experimental data becomes available [27]. Further refinement of the models may be possible using new modelling methods such as D-I-TASSER [83] and trROSETTA [84–87]. These methods use different approaches and implementations of the standard AF2M. It is also important to acknowledge the current uncertainty regarding AlphaFold2-Multimer’s suitability in predicting splice variants.

For the MD simulations, simple membranes (composed of POPC and cholesterol molecules) were used rather than complex membrane systems that more accurately replicate the composition of human epithelial membranes [64]. The simpler membrane was deemed appropriate for this study as the receptor rather than the membrane characteristics were being studied. Further studies should investigate the receptor stability and interactions with the membrane environment in different membrane systems to account for the variability in membrane composition throughout the human body [64] as well as the crucial modulatory effect of membrane cholesterol on P2X7R function [88].

#### Conclusion and future direction

In the present study we have evaluated the suitability of the deep learning approach AF2M in predicting the structure of large purinergic receptor protein complexes, specifically the P2X7R. The developed protocol was used to generate models of P2X7R complexes incorporating alternatively spliced subunits together with wild type subunits in 1:2 and 2:1 stoichiometric ratio. The models were validated using AF2M confidence metrics and further supported by MD simulation results which showed that the generated models were overall stable over time.

The present study provides the first insight into the structure of P2X7Rs that incorporate alternatively spliced subunits. Precision medicine is an emerging model for treating patients with conditions that are clinically and biologically heterogeneous and therefore require individualised treatment. We have developed a model pipeline for analysis of binding of known antagonists and novel molecules to P2X7R alternatively spliced isoforms that have the potential to be translated into treatments for cancers, inflammatory conditions, trauma, and neurodegenerative diseases.

## Data Availability

AF2M models are available as part of this submission as supporting material.
